# Bioinformatics analysis of the expression and role of microRNA-221-3p in head and neck squamous cell carcinoma

**DOI:** 10.1186/s12885-021-08039-5

**Published:** 2021-04-12

**Authors:** Ziyan Zhou, Wenling Wu, Jixi Li, Chang Liu, Zixi Xiao, Qinqiao Lai, Rongxing Qin, Mingjun Shen, Shuo Shi, Min Kang

**Affiliations:** 1 Department of Radiation Oncology, Guangxi Medical University First Affiliated Hospital, Nanning, 530021 Guangxi People’s Republic of China; 2 Guangxi Tumor Radiation Therapy Clinical Medical Research Center, Nanning, 530021 Guangxi People’s Republic of China; 3 Department of Medical Oncology, The First Affiliated Hospital of Guangxi Medical University, Nanning, 530021 Guangxi People’s Republic of China; 4 Guangxi Medical University, Nanning, 530021 Guangxi People’s Republic of China; 5 Department of Thoracic Surgery, The First Affiliated Hospital of Guangxi Medical University, Nanning, Guangxi Zhuang Autonomous Region People’s Republic of China

**Keywords:** HNSCC, miR-221-3p, RT-qPCR, TCGA, Bioinformatics

## Abstract

**Background:**

Head and neck squamous cell carcinoma (HNSCC) is the sixth most common cancer worldwide, associated with a high rate of morbidity and mortality. However, the target genes of miR-221-3p and the underlying mechanism involved in HNSCC are still not clear. Therefore, in the current study, we studied the role of miR-221-3p in the HNSCC.

**Methods:**

Tissues collected from 48 control and 21 HNSCC patients were processed to check the differential expression of miR-221-3p by RT-qPCR. Overexpression of microRNA-221-3p (miR-221-3p) is significantly correlated to the onset and progression of HNSCC. We also conducted the meta-analysis of the cancer literature from the cancer genome atlas (TCGA) and the Gene Expression Omnibus (GEO) database to estimate the expression of miR-221-3p in HNSCC. The miR-221-3p target genes in the HNSCC were predicted with the miRWalk and TCGA databases, and functionally annotated via the Gene Ontology. Finally, Spearman’s analysis was used to determine the role of the related target genes in important pathways involved in the development of HNSCC.

**Results:**

We observed a significantly higher expression of miR-221-3p in HNSCC compared to the normal with a summary receiver operating characteristic (sROC) of 0.86(95% Cl: 0.83,0.89). The KEGG and GO comprehensive analysis predicted that miR-221-3p might be involved in the development of HNSCC through the following metabolic pathways, viz. Drug metabolism - cytochrome P450 UGT1A7 and MAOB may be important genes for the role of miR-221-3p.

**Conclusion:**

Based on bioinformatics analysis, our results indicate that miR-221-3p may be used as a non-invasive and hypersensitive biomarker in the diagnosis. Thus, it can be concluded that miR-221-3p may be an extremely important gene locus involved in the process of the deterioration and eventual tumorigenesis of HNSCC. Hopefully, additional work will validate its usefulness as a target for future clinical research.

## Background

Head and neck squamous cell carcinoma (HNSCC), encompassing oral squamous cell carcinoma, oropharyngeal squamous cell carcinoma, hypopharyngeal squamous cell carcinoma and laryngeal squamous cell carcinoma, is the sixth most common cancer worldwide, accounting for approximately 1–2% of all cancer deaths, with approximately 600,000 new cases detected each year globally [1–3]. The treatment of patients at the early stage is relatively successful, but the overall survival rate of recurrent or metastatic HNSCC remains low and has barely seen any improvements for decades [4, 5]. Although the past three decades have seen several improvements in diagnostic tools and treatment regimens, the overall survival rate for advanced (stage III-IV) HNSCC is only 65% [6, 7]. The current standard of care for such patients is surgery along with radiation and chemotherapy, but these have not significantly improved the 5-year survival of HNSCC patients. The main reason for the decline in patient survival is the lack of effective therapeutic targets for the development of HNSCC [8, 9].

Recent years have seen an increasing concentration of research on microRNAs (miRNAs) [10, 11], which are a type of highly conserved single-stranded noncoding RNA containing 17 ~ 22 nucleotides and are involved in the process of tumorigenesis, cell survival, and chemosensitivity [12–14]. They bind to the 3′ untranslated region (3’UTR) of different target mRNA (messenger RNA) genes to degrade or inhibit the mRNA translation of target genes associated with a tumor suppressor function [13, 15, 16]. In normal healthy individuals, miR-221-3p is observed to play a role in the process of vascular proliferation [17], while the tumor promoter microRNA-221 is involved in the process of regulating apoptosis of tumor cells [18–20] and is associated with a variety of cancer types, including hepatocellular cancer [21], cutaneous melanoma [19], prostate cancer [20], and non-small cell lung cancer [22].

Studies have shown that specific miRNA profiles can be identified between tumor tissue and adjacent healthy tissue in HNSCC patients [2, 23]. For instance, studies have reported a link between miR-221 and vascular invasion in HNSCC [14, 15, 24–26]. However, the definite target gene of miR-221-3p and its biological mechanism of action are still not clear. Thus, in the current study, we investigated the expression of miR-221-3p in HNSCC and attempted to explore the correlation between the two.

## Methods

### RT -qPCR

Tissue specimens were collected from 48 control and 21 HNSCC patients from the Department of Pathology, First Affiliated Hospital of Guangxi Medical University (Nanning, Guangxi Medical University). Total RNA was extracted from formalin-fixed, paraffin-embedded (FFPE) tissues using an FFPE RNA kit (Omega Bio-Tek, Norcross, GA, USA). RNA was reverse-transcribed into cDNA using the Mir-X miRNA qRT-PCR SYBR Kit (Takara, California, USA). The cDNA samples were processed for qPCR with SYBR-Green Master Mix (Takara, Tokyo, Japan) on an ABI 7500 cycler (Applied Biosystems) under the following conditions: initial denaturation at 95 °C for 30 s, followed by 40 cycles of denaturation at 95 °C for 5 s and annealing at 60 °C for 34 s. The expression of miR-221-3p in HNSCC tissues relative to negative control (NC) tissues was calculated using the 2- ΔΔCT method, with U6 as the internal control. The primers for miR-221-3p and U6 were synthesized by TaKaRa (Dalian, Liaoning, China), and the sequences were as follows: miR-221-3p: forward 5′-AGCUACAUUGUCUGCUGGGUUUC − 3′ and reverse 5′ mRQ 3′; U6: forward 5′-GGAACGATACAGAGAAGATTAGC-3′ and reverse 5′-TGGAACGCTTCACGAATTTGCG-3′. All the experiments were repeated three times.

### Literature search and selection strategy

A literature search was conducted in PubMed, Chinese Biomedical Literature Database (CBM), Science Direct, China National Knowledge Infrastructure (CNKI) database, Web of Science, Wiley Online Library, EMBASE, China Science and Technology Journal Database (VIP) and Wanfang Database for this study. We searched the databases from the earliest available data to October 1, 2019. The following keywords were used: (HNSCC OR SCC OR “squamous cell cancer” OR “squamous cell carcinoma”) AND (oropharynx OR oropharyngeal OR “head and neck” OR nose OR nasopharynx OR “nasal sinus” OR “nasal cavity” OR “oral cavity” OR hypopharynx OR oral OR laryngopharynx OR larynx OR laryngopharyngeal OR laryngeal OR pharyngeal OR tongue OR tonsil OR tonsillar OR cheek OR palatal OR “paranasal sinuses” OR buccal OR lip) AND (microRNA-221-3p OR miRNA-221-3p OR “miR 221-3p” OR “miRNA 221-3p” OR miRNA221-3p OR miR221-3p).

### Selection criteria and data extraction

The databases were searched independently by two researchers who selected the studies based on the following inclusion criteria: (1) comparison of HNSCC and noncancerous tissues; (2) validation of miR-221-3p expression levels via reverse transcription quantitative PCR (RT-qPCR); (3) evaluation of the association between miR-221-3p expression and clinical outcomes; and (4) availability of sufficient data to calculate the mean, standard deviation (SD) and 95% confidence intervals (95% CIs). The articles were eliminated if they met any of the following exclusion criteria: (1) irrelevant to the research focus; (2) inclusion of unqualified data; (3) publication language other than English or Chinese; (4) overlapping or duplicate publications; and (5) letters, reviews, comments, editorials, conference articles, laboratory studies or case reports. The reviewers independently appraised the quality of data in each eligible study and extracted the first author name, year of publication, country of origin, sample type, sample size and analysis method. For articles with incomplete information, the authors were contacted to obtain relevant information.

### Microarray data collection from GEO

The microarray data of HNSCC samples uploaded until October 1, 2019, were obtained from the GEO database (https://www.ncbi.nlm.nih.gov/gds/) using the following keywords: (HNSCC OR SCC OR “squamous cell cancer” OR “squamous cell carcinoma”) AND (oropharynx OR oropharyngeal OR “head and neck” OR nose OR nasopharynx OR “nasal sinus” OR “nasal cavity” OR “oral cavity” OR hypopharynx OR oral OR laryngopharynx OR larynx OR laryngopharyngeal OR laryngeal OR pharyngeal OR tonsil OR tonsillar OR tongue OR cheek OR palatal OR “paranasal sinuses” OR buccal OR lip) AND (microRNA OR miRNA OR “miR” OR “miRNA”). The inclusion criteria for the microarray datasets were as follows: (1) comprising data from HNSCC and noncancerous tissues; (2) evaluation of the association between miR-221-3p expression and clinical outcomes; and (3) availability of sufficient data to calculate the mean, SD and 95% CI. The exclusion criteria were as follows: (1) unrelated to this study; (2) unqualified data; and (3) publication language other than English.

### RNA sequencing data selection from TCGA

The miR-221-3p expression data of HNSCC and normal tissues and the clinicopathological parameters of patients were downloaded and extracted from the OncoLnc website (http://www.oncolnc.org/).

### Predicting the target genes of miR-221-3p in HNSCC

We use the MiRWalk 2.0 [27] (http://zmf.umm.uniheidelberg.de/apps/zmf/mirwalk2/) and GEPIA (http://gepia.cancer-pku.cn/index.html) databases to retrieve differentially expressed genes of miR-221-3p in HNSCC. Gene Ontology (GO) [28] analysis, which was used to define the biological processes (BPs), molecular functions (MFs), and cellular components (CCs) of the target genes, and Kyoto Encyclopedia of Genes and Genomes (KEGG) [29] pathway enrichment analyses were conducted using R (version 3.6.1), and the ClusterProfiler package of R was used to visualize the results. The Search Tool for the Retrieval of Interacting Genes (STRING) (https://string-db.org/) was then used to establish a protein-protein interaction (PPI) network of the miR-221-3p target genes associated with the significantly enriched pathways. The expression levels of the miR-221-3p target genes in HNSCC and nontumor tissues were determined with UALCOULD [30] (http://ualcan.path.uab.edu/index.html). Finally, the LinkedOmics [31] (http://www.linkedomics.org/) Spearman’s analysis tool was applied to determine the correlation between the expression levels of miR-221-3p and the potential target genes involved in notable signaling pathways.

### Statistical analysis

Independent-Samples T Test for the RT-qPCR analysis of two groups. SPSS 24.0 software (IBM, Somers, NY) was used for the statistical analysis. The mean ± SD (X ± s) were calculated, and t test was used for comparisons between groups for all the measurement data. Data that were used for our meta-analysis as well as TCGA data (Nanning, Guangxi Zhuang Autonomous Region, China) were analyzed using STATA 15.0 software. The standard mean difference (SMD) and 95% confidence interval were used to estimate the expression value of miR-221-3p. Scatter plots of the data of both the normal and HNSCC tissues were prepared with GraphPad Prism 6 (Nanning, Guangxi Zhuang Autonomous Region, China) and used to assess the expression level of miR-221-3p. Evidence of bias was assessed by visual funnel plots and Egger’s regression asymmetry test. In addition, the I^2^ index was used to evaluate the potential heterogeneity of the selected data. When I^2^ > 50% or *P* < 0.05, we used the fixed effect model; otherwise, the random effect model was used.. The count rate was expressed as a percentage (%), and we used the X^2^ test for comparisons. The rates of true positives (TPs), false positives (FPs), false negatives (FNs) and true negatives (TNs) were used to determine the diagnostic value of miR-221-3p. We used Meta-disc software to calculate the specificity, sensitivity, negative and positive likelihood ratios, and summary receiver operating characteristic (sROC) curve. We also performed sensitivity analysis to assess the differences between the sample sizes. Moreover, all the miR-221-3p expression data, which included the TCGA sequencing data, were normalized to log2 to improve the normality of the measurements. *P* < 0.05 was taken as a statistically significant difference for all analyses.

## Result

### miR-221-3p is highly expressed in the tumor tissues

**Fig. 1:**
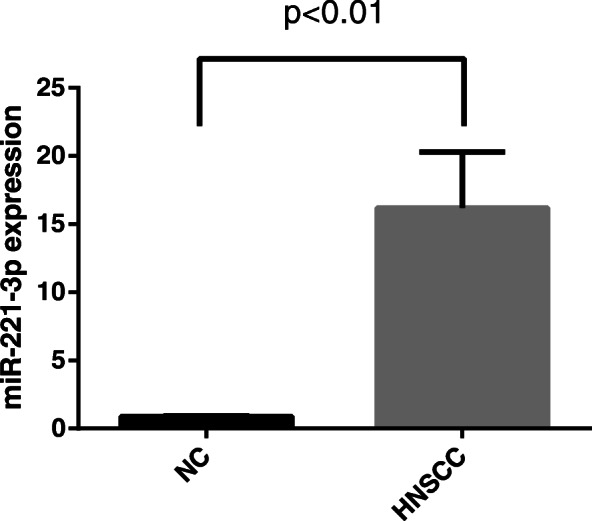
MiR-221-3p levels in HNSCC and NC tissue specimens. RT-qPCR was used to detect miR-221-3p was significantly up-regulated in the tumor tissues of HNSCC (*n* = 21) relative to control tissues (*n* = 48)(p<0.01)

The RT-qPCR data clearly showed that miR-221-3p is highly expressed in the tissues of nasopharyngeal carcinoma patients compared to the controls (*p* < 0.05) (Fig.1).

### Selection of relevant literature and microarray data extraction

**Fig. 2:**
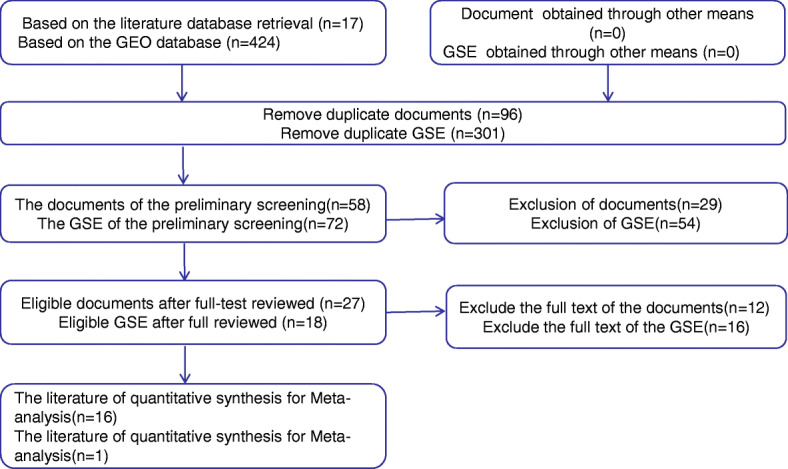
The flow chart of literature search and selection of relevant studies. Sixteen out of 424 GEO microarray datasets met our inclusion criteria. At the same time, a total of 17 articles were retrieved from the initial search, and one papers (Zhou Cheng. et al) were selected after the full-text review based on the inclusion criteria

A total of 17 articles were retrieved from the initial search, and the following paper was selected after the full-text review based on the inclusion criteria: Zhou Cheng. et al. (PMID:30928631) [32] (Fig.2). Sixteen out of 424 GEO microarray datasets, including GSE11163, GSE103931, GSE107591, GSE113956, GSE28100, GSE31277, GSE34496, GSE45238, GSE51829, GSE58911, GSE69002, GSE70289, GSE73171, GSE75630, GSE82064, and GSE98463, met our inclusion criteria. Information for each included dataset is summarized in Table 1.

**Table 1:** The main features of included studies for this meta-analysis

Researcher	Year	Country	Cancer/normal	Methods	Sample
TCGA	2019	USA	523/44	qPCR	Tissue
GSE11163	2012	USA	16/5	qPCR	Tissue
GSE103931	2018	Taiwan	30/19	qPCR	Tissue
GSE107591	2018	USA	24/23	qPCR	Tissue
GSE113956	2019	China	25/15	qPCR	Tissue
GSE28100	2015	USA	17/3	qPCR	Tissue
GSE31277	2018	Brazil	15/15	qPCR	Tissue
GSE34496	2017	USA	44/25	qPCR	Tissue
GSE45238	2019	USA	40/40	qPCR	Tissue
GSE51829	2013	China	4/4	qPCR	Tissue
GSE58911	2018	USA	15/15	qPCR	Tissue
GSE69002	2017	USA	3/4	qPCR	Tissue
GSE70289	2018	USA	12/4	qPCR	Tissue
GSE73171	2017	Hong Kong	3/3	qPCR	Tissue
GSE75630	2016	Australia	28/18	qPCR	Tissue
GSE82064	2017	Switzerland	78/18	qPCR	Tissue
GSE98463	2018	Spain	8/8	qPCR	Tissue
Zhou Cheng. et al	2019	China	26/26	qPCR	Tissue

**Fig. 3:**
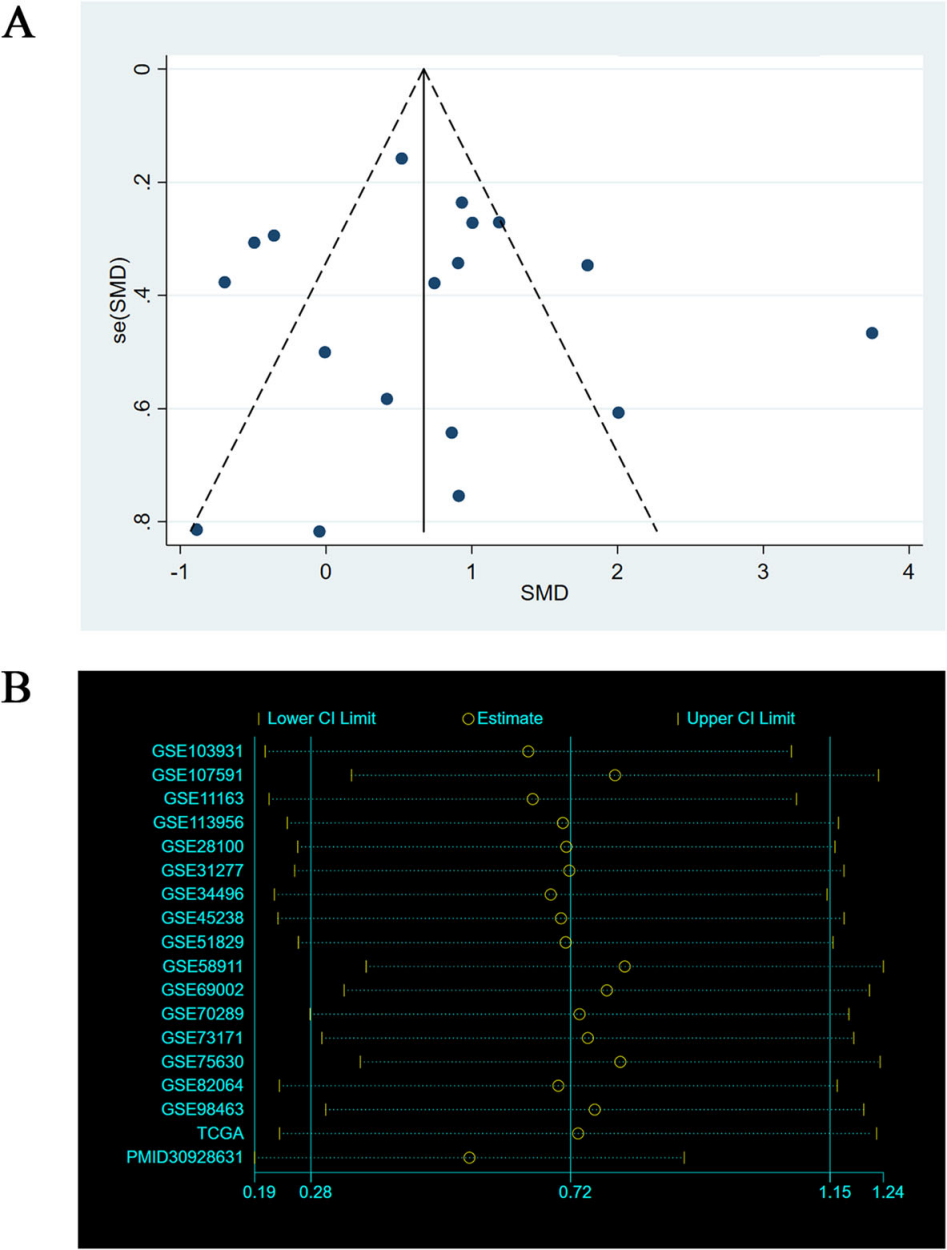
Bias analysis. **a** Funnel plots of published data bias for meta-analysis of miR-21. **b** Sensitivity analysis for meta-analysis of miR-221-3p

**Fig. 4:**
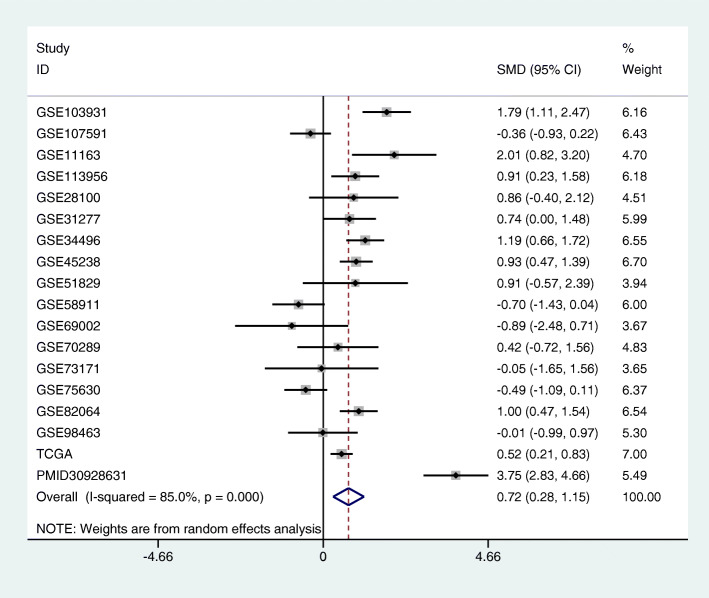
Forest plots. The combined SMD of miR-221-3p expression levels in HNSCC samples was 0.717(95% Cl:0.283, 1.152)

**Fig. 5:**
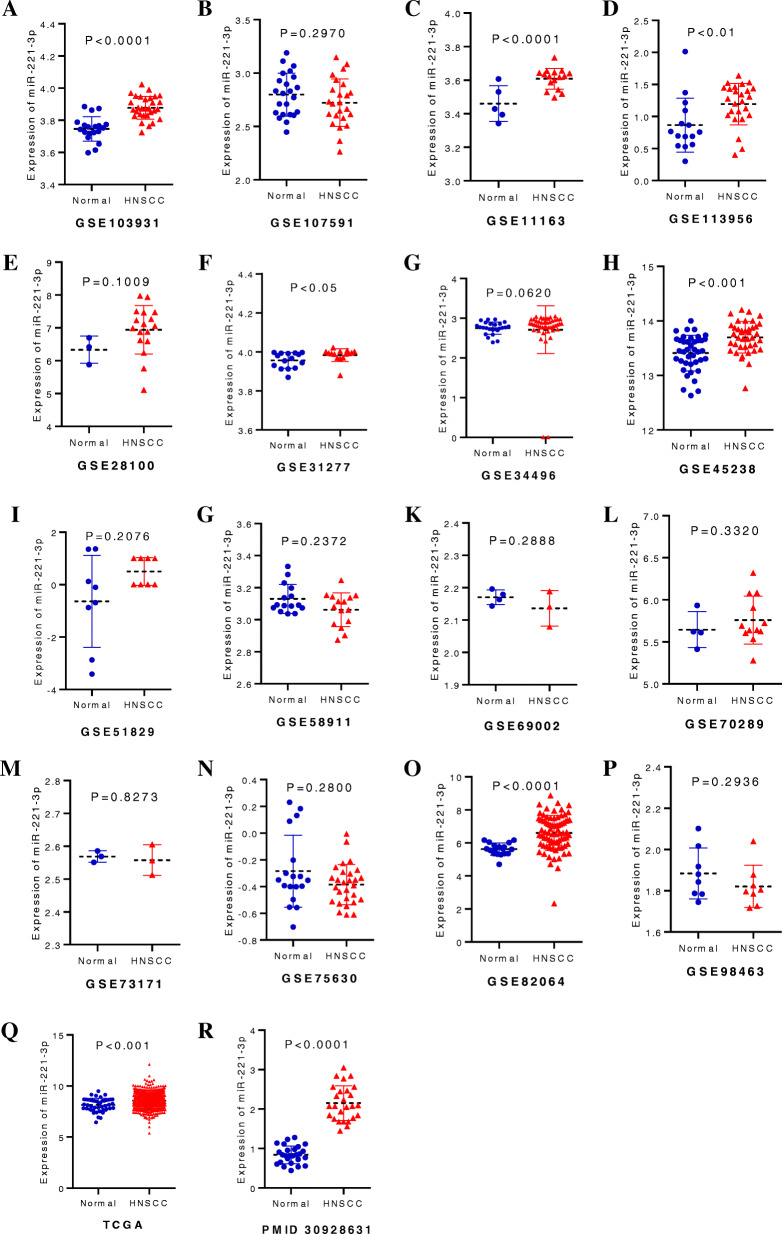
The expression data of miR-221-3p in HNSCC from TCGA, each included GEO and the literature

Risk of bias assessment and meta-analysis of miR-221-3p in HNSCThe results of the visual funnel plots show a symmetrical shape, which indicates the absence of any significant publication bias as a whole. Sensitivity analysis performed to assess the heterogeneity of the samples showed that there were no significant differences between the studies (Fig.3). There were 911 HNSCC samples and 289 normal samples from the PubMed, GEO, and TCGA datasets. The meta-analysis performed on all data to explore the expression level of miR-221-3p in HNSCC indicated a high degree of heterogeneity between these studies. Therefore, the random-effects model was selected, and the combined standard mean difference (SMD) was observed to be 0.72 (95% CI: 0.28, 1.15) (Fig.4). The meta-analysis data indicate that miR-221-3p expression is significantly upregulated in HNSCC, which is consistent with the results obtained by RT-qPCR. In addition, miR-221-3p expression was significantly increased in HNSCC in the GSE11163, GSE103931, GSE113956, GSE31277, GSE45238, GSE82064, TCGA and PMID30928631 datasets, and the expression levels of miR-221-3p in HNSCC and normal tissues from each included dataset are shown in Fig.5.

### Diagnostic value of miR-221-3p in HNSCC

**Fig. 6:**
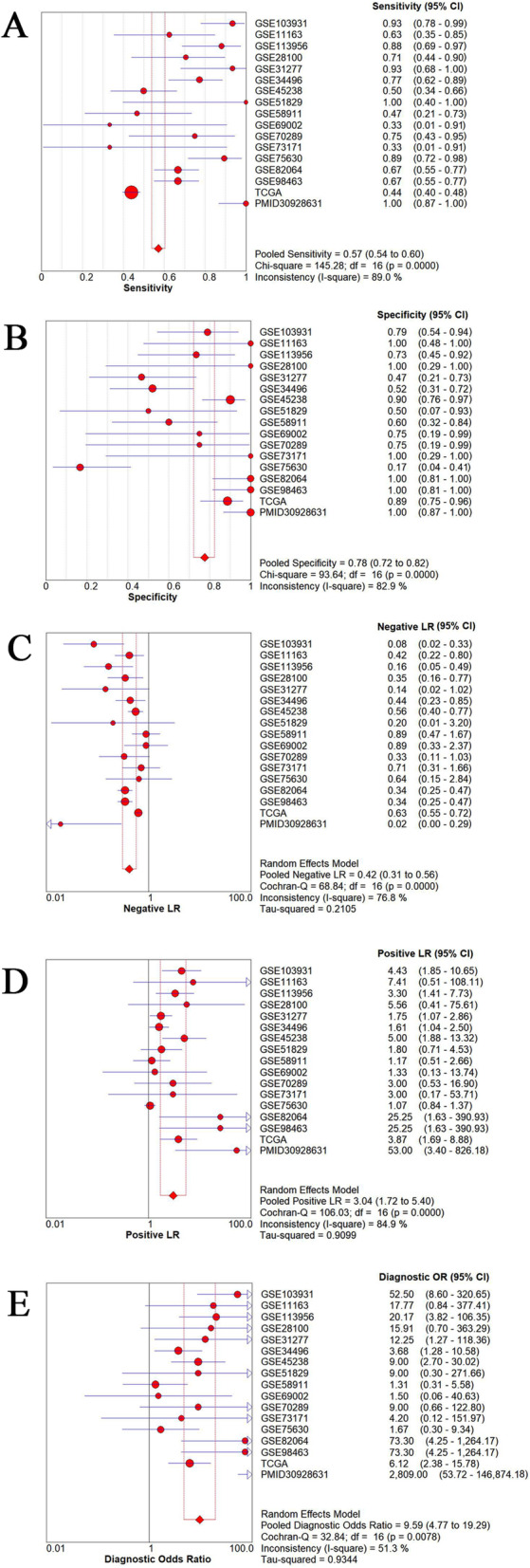
The diagnostic value of miR-221-3p in HNSCC. **a** Sensitivity; (**b**) Specificity; (**c**) Negative LR; (**d**) Positive LR; (**e**) Diagnostic odds ratio

**Fig. 7:**
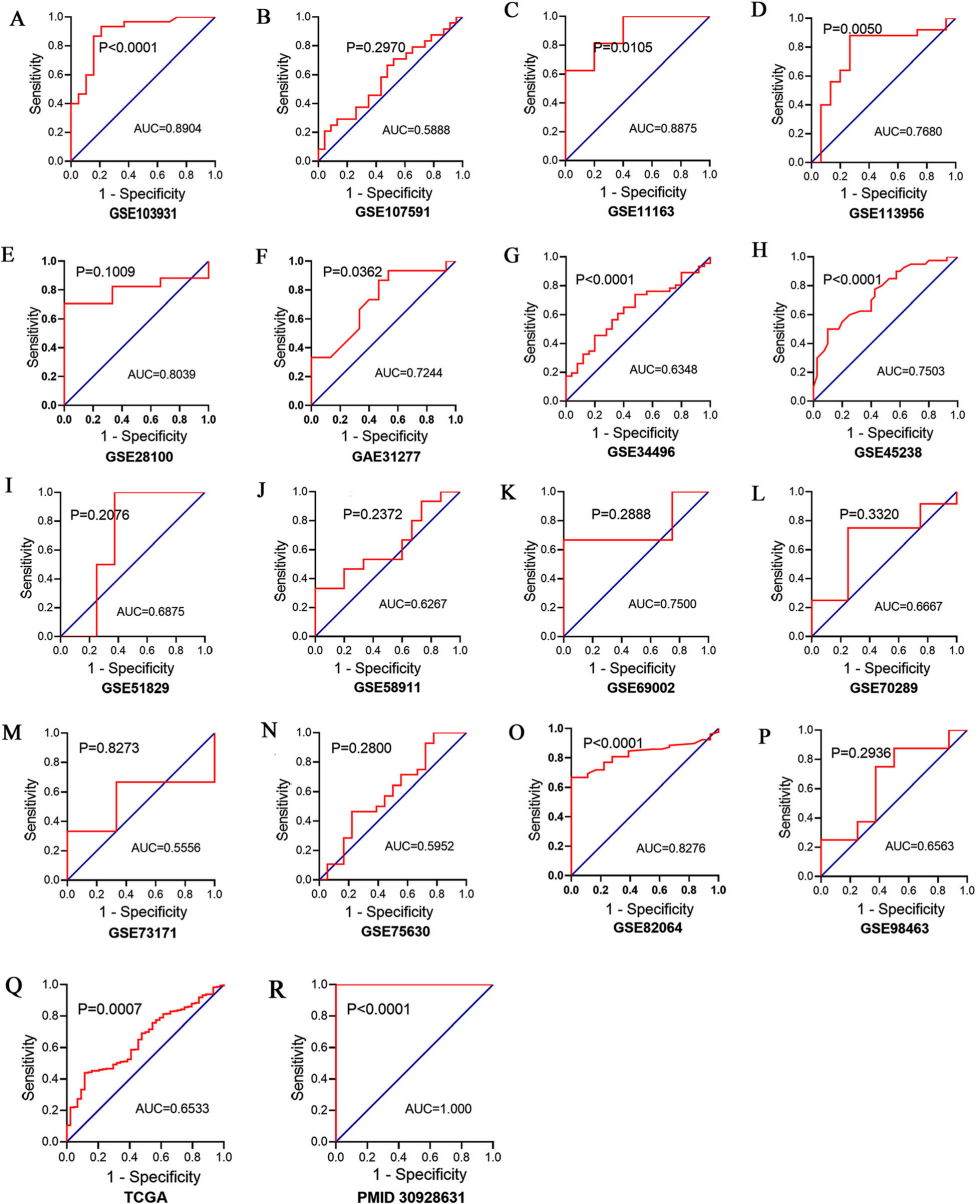
Receiver operating characteristic (ROC) curves showing the diagnostic value of miR-221-3p in HNSCC

**Fig. 8:**
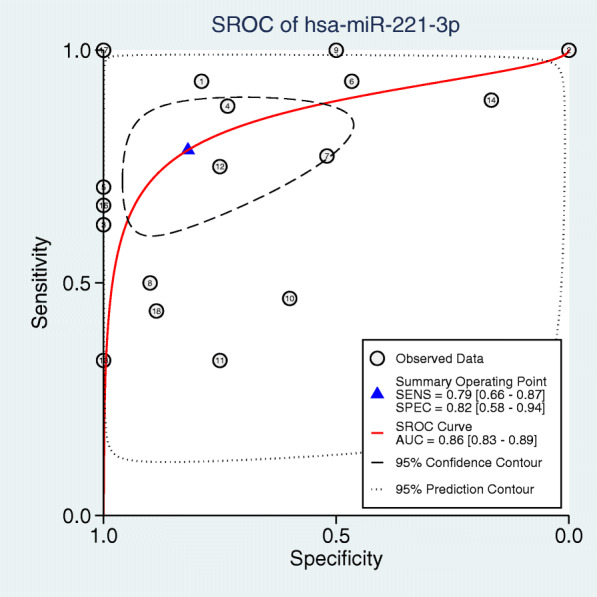
Summary ROC (SROC) curve showing the diagnostic performance of miR-221-3p in HNSCC. The sROC curve AUC was 0.86 (95% CI: 0.83–0.89), indicating high predictive power

The random-effects model used to analyze the diagnostic value of miR-221-3p for HNSCC showed significant heterogeneity in the likelihood ratio (negative and positive) sensitivity and specificity analyses. The diagnostic meta-analysis results indicated that the pooled specificity, sensitivity, likelihood ratio (negative and positive) and diagnostic odds ratio were 0.78 (95% CI: 0.72–0.82), 0.57 (95% CI: 0.54–0.60), 0.42 (95% CI: 0.31–0.56) and 3.04 (95% CI: 1.72–5.40), 9.59(95% CI: 4.77–19.29) respectively (Fig.6). The receiver operating characteristic (ROC) curve of GSE103931, GSE11163, GSE113956, GSE31277, GSE34496, GSE45238, GSE82064, TCGA and PMID30928631 is statistically significant (*p* < 0.05, Fig.7). Based on the ROC curve of each study, the overall ROC curve indicated that the area under the sROC curve (AUC) was 0.86 (95% Cl: 0.83, 0.89) (Fig.8).

### Clinical features of HNSCC based on the TCGA

Based on the HNSCC sample data extracted from the TCGA database, we analyzed 527 samples, and the results indicated the absence of any statistically significant difference between miR-221-3p expression and age, lymphatic invasion, neoplasm histologic grade and any other clinicopathological features. However, the results indicated statistically significant differences in the expression of miR-221-3p in different tissues, sexes, and even alcohol consumption statuses (Table 2).

**Table 2:** The correlation between miR-221-3p expression levels and clinic characteristics based on TCGA database

Clinicopathological features	Terms	n	Mean ± SD	*p*-value
Unpaired tissue	Normal	44	8.16 ± 0.64	0.001
	HNSCC	480	8.55 ± 0.78	
Sex	Male	383	8.45 ± 0.79	0.001
	Female	141	8.71 ± 0.68	
Age	< 60	234	8.50 ± 0.76	0.559
	> = 60	290	8.54 ± 0.78	
lymphovascular invasion	NO	232	8.54 ± 0.81	0.429
	YES	292	8.49 ± 0.75	
Neoplasm histologic grade	G1-G2	371	8.49 ± 0.73	0.260
	G3-G4	156	8.58 ± 0.82	
M stage	M0	498	8.51 ± 0.78	0.967
	M1	29	8.51 ± 0.57	
N stage	N0	246	8.53 ± 0.76	0.572
	N1-N3	281	8.49 ± 0.77	
Alcohol	NO	164	8.62 ± 0.76	0.047
	YES	363	8.48 ± 0.76	
HPV status	Negative	194	8.41 ± 0.81	0.066
	Positive	330	8.55 ± 0.81	
Perineural invasion	NO	198	8.53 ± 0.81	0.693
	YES	329	8.50 ± 0.80	

Notes: HNSCC, Head and neck squamous cell carcinoma; T stage, size or direct extent of the primary tumor; N stage, degree of spread to regional lymph nodes; M stage, presence of distant metastasis; SD, standard deviation

### The prospective target genes of miR-221-3p in HNSCC

**Fig. 9:**
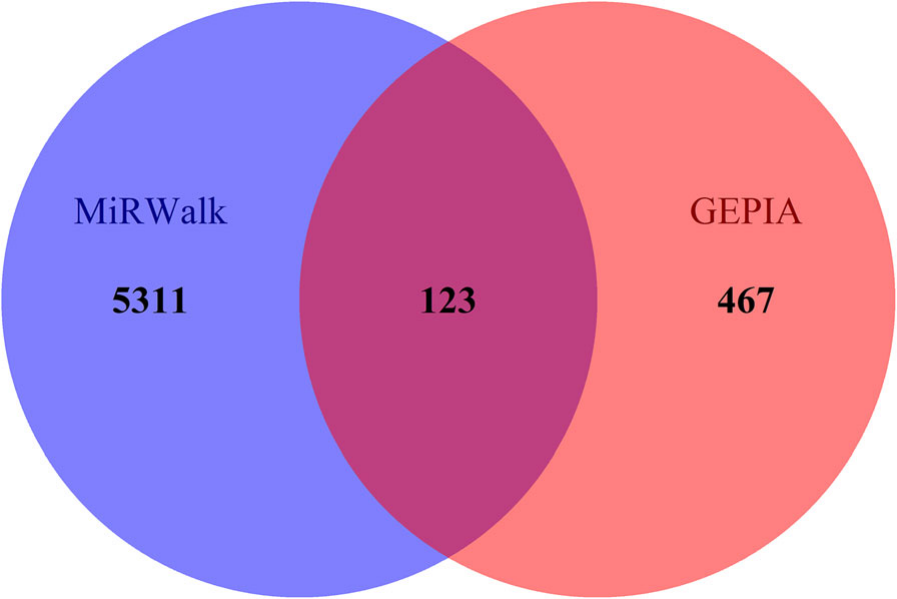
Venn diagram showing overlapping miR-221-3p target genes

**Fig. 10:**
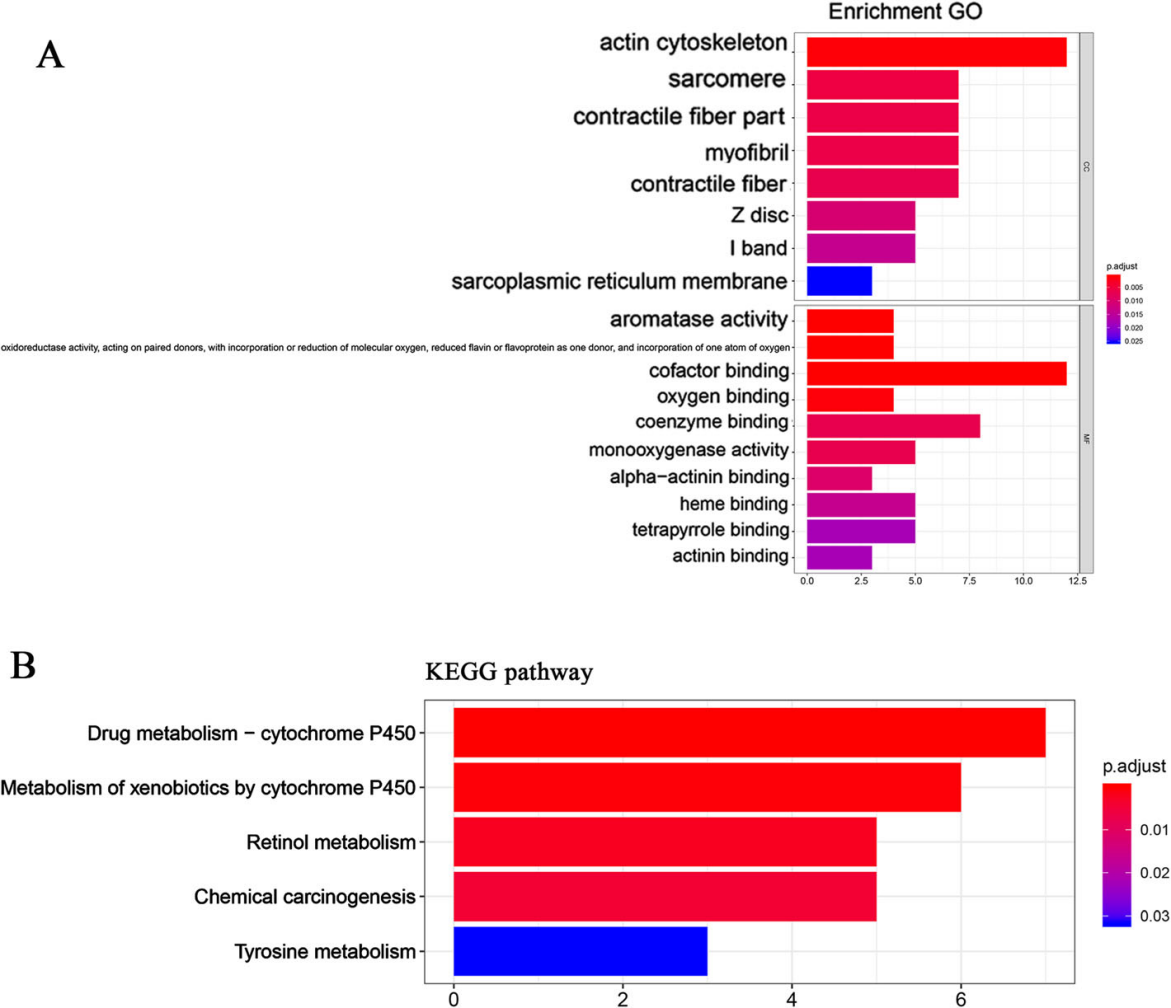
Enrichment analysis of GO and KEGG pathways of miR-221-3p target genes. **a** Gene ontology (GO) of candidate genes; (**b**) Kyoto Encyclopedia of Genes and Genomes (KEGG) analysis

**Fig. 11:**
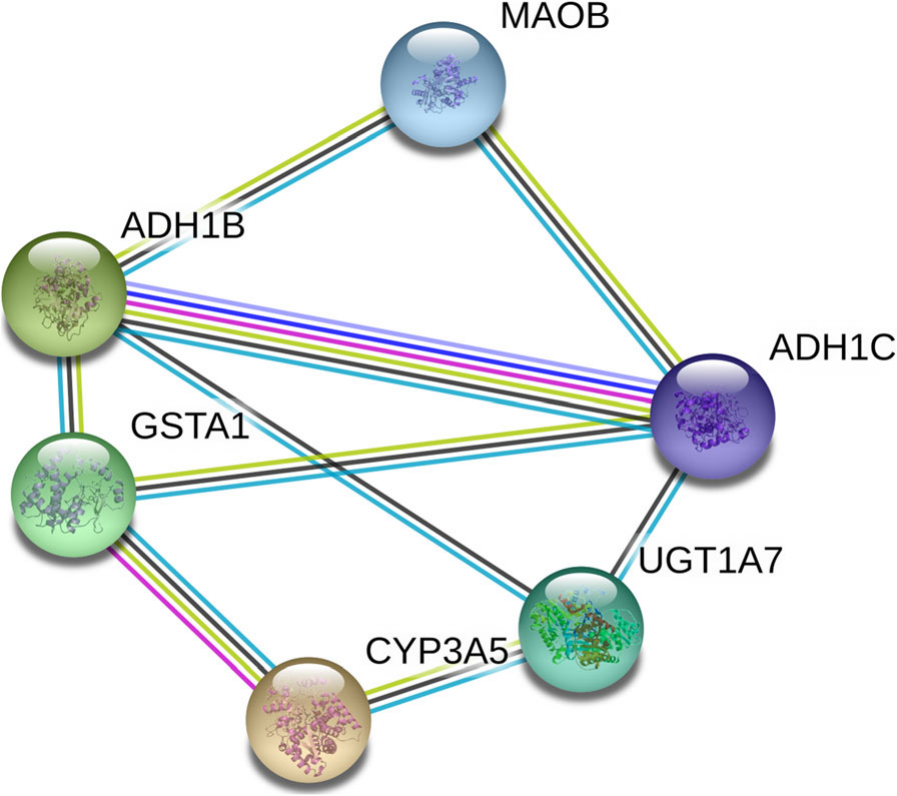
PPI network. The nodes represent target genes and the lines are associations between genes, which showing the connections among the seven miR-221-3p targets associated with the Drug metabolism-cytochrome P450 pathway

By using the MiRWalk 2.0 and GEPIA databases, we retrieved 5311 and 467 differentially expressed genes, respectively. Bioinformatics analysis showed a total of 117 overlapping genes that were involved (Fig.9, Table 3). These overlapped 117 genes could be considered as the target genes that miR-221-3p might play a role in HNSCC. The enriched GO and KEGG pathway categories of the overlapped 117 genes with *p* < 0.05 are shown in Fig.10, Table 4 and Table 5. For the cellular components, the identified target genes were mostly enriched in the actin cytoskeleton, sarcomeres, and contractile fiber part; for the molecular functions, the target genes were mainly enriched in aromatase activity, oxidoreductase activity, and cofactor binding. KEGG enrichment analysis showed that miR-221-3p plays a significant role in HNSCC through a variety of pathways, including the drug metabolism pathways of the cytochrome P450 signaling pathway. The drug metabolism-cytochrome P450-related genes include GSTA1, UGT1A7, CYP3A5, FMO2, MAOB, ADH1C and ADH1B, and the protein-protein interaction (PPI) network of these genes is shown in Fig.11.

**Table 3:** The promising target genes of miR-221-3p in HNSCC with TCGA and MiRWalk

Sources of genes	Genes
TCGA and MiRWalk	AADAC, ACPP, ADH1B, ADH1C, ALDH1A1, AMOT, ANKRD35, ANXA1, AQP3, ASB5, ATP2A1, BCAS1, C2orf54, C7, CA3, CAB39L, CAPN14, CAPN5, CAPN6, CD24, CEACAM6, CEACAM7, CGNL1, CHRDL1, CLU, COBL, CP, CRISP3, CRYM, CXCL17, CXCR2, CYP2F1, CYP3A5, CYP4B1, CYP4X1, DEPTOR, EHF, EIF1AY, EMP1, ENDOU, FAM189A2,FAM3D, FMO2, FOXA1, FUT3, GABRP, GATM, GBP6, GCNT3, GGT6, GPD1, GPT2, GPX3, GSTA1, HLF, HMGCS2, HPGD, HSPB8, IL1RN, KALRN, KLHL41, KRT23, KRT78, LDB3, LYNX1, MANSC1, MAOB, MAPT, MB, METTL7A, MGLL, MUC21, MUC4, MYL2, MYZAP, NCCRP1, NDRG2, NFIX, PADI1, PADI2, PAX9, PCP4L1, PDK4, PEBP4, PI16, PLEKHA6, PPP1R1A, PPP1R3C, PSCA, PTN, RAET1E, RNF222, RRAGD, SCIN, SELENBP1, SERPINB13, SFRP1, SH3BGRL2, SLC16A7, SLC5A1, SMPX, SORBS1, SORBS2, STEAP4, SYNPO2, THSD4, TMEM45B, TNNI1, TPRG1, TRDN, TTC9, UGT1A7, UPK1A, VSIG10L, XIRP2, ZBTB7C, ZSCAN18

Notes: HNSCC: Head and neck squamous cell carcinoma; TCGA: The Cancer Genome Atlas

**Table 4:** Predictive target genes of miR-221-3p with GO analysis

GO ID	GO term	Count (%)	Gene symbol	*p*-value
**Cellular component**				
GO:0015629	actin cytoskeleton	12	AMOT, ANXA1, CGNL1, COBL, KALRN, MYL2,MYZAP, SCIN, SORBS1, SORBS2, SYNPO2, XIRP2	6.22E-06
GO:0030017	sarcomere	7	KLHL41, LDB3, MYL2, MYZAP, SORBS2, SYNPO2, XIRP2	6.06E-05
GO:0044449	contractile fiber part	7	KLHL41, LDB3, MYL2, MYZAP, SORBS2, SYNPO2, XIRP2	1.12E-04
GO:0030016	myofibril	7	KLHL41, LDB3, MYL2, MYZAP, SORBS2, SYNPO2, XIRP2	1.32E-04
GO:0043292	contractile fiber	7	KLHL41, LDB3, MYL2, MYZAP, SORBS2, SYNPO2, XIRP2	1.90E-04
GO:0030018	Z disc	5	LDB3, MYZAP, SORBS2, SYNPO2, XIRP2	3.44E-04
GO:0031674	I band	5	LDB3, MYZAP, SORBS2, SYNPO2, XIRP2	5.21E-04
GO:0033017	sarcoplasmic reticulum membrane	3	ATP2A1, KLHL41, TRDN	1.11E-03
**Molecular function**				
GO:0070330	aromatase activity	4	CYP2F1, CYP3A5, CYP4B1, CYP4X1	6.04E-06
GO:0016712	oxidoreductase activity, acting on paired donors, with incorporation or reduction of molecular oxygen, reduced flavin or flavoprotein as one donor, and incorporation of one atom of oxygen	4	CYP2F1, CYP3A5, CYP4B1, CYP4X1	8.86E-06
GO:0048037	cofactor binding	12	ALDH1A1, CRYM, CYP3A5, CYP4B1, CYP4X1, FMO2, GPD1, GPT2, HPGD, MAOB, MB, STEAP4	9.13E-06
GO:0019825	oxygen binding	4	CYP2F1, CYP3A5, CYP4B1, MB	1.73E-05
GO:0050662	coenzyme binding	8	ALDH1A1, CRYM, FMO2, GPD1, GPT2, HPGD, MAOB, STEAP4	1.30E-04
GO:0004497	monooxygenase activity	5	CYP2F1, CYP3A5, CYP4B1, CYP4X1, FMO2	1.46E-04
GO:0051393	alpha-actinin binding	3	LDB3, SYNPO2, XIRP2	2.37E-04
GO:0020037	heme binding	5	CYP3A5, CYP4B1, CYP4X1, MB, STEAP4	3.88E-04
GO:0046906	tetrapyrrole binding	5	CYP3A5, CYP4B1, CYP4X1, MB, STEAP4	5.87E-04
GO:0042805	actinin binding	3	LDB3, SYNPO2, XIRP2	6.30E-04
GO:0016705	oxidoreductase activity, acting on paired donors, with incorporation or reduction of molecular oxygen	5	CYP2F1, CYP3A5, CYP4B1, CYP4X1, FMO2	1.24E-03
GO:0033130	acetylcholine receptor binding	2	LYNX1, PSCA	1.47E-03
GO:0016722	oxidoreductase activity, oxidizing metal ions	2	CP, STEAP4	2.14E-03
GO:0030674	protein binding, bridging	5	MAPT, SORBS1, SORBS2, SYNPO2, TRDN	2.20E-03

Notes: GO: Gene Ontology

**Table 5:** KEGG pathway of validated target genes of miR-221-3p

KEGG ID	KEGG term	Count (%)	Gene symbol	*p-*value
hsa00982	Drug metabolism - cytochrome P450	7	GSTA1, UGT1A7, CYP3A5, FMO2, MAOB, ADH1C, ADH1B	3.18E-07
hsa00980	Metabolism of xenobiotics by cytochrome P450	6	GSTA1, UGT1A7, CYP3A5, CYP2F1, ADH1C, ADH1B	8.10E-06
hsa00830	Retinol metabolism	5	UGT1A, ALDH1A1, CYP3A5, ADH1C, ADH1B	6.43E-05
hsa05204	Chemical carcinogenesis	5	GSTA1, UGT1A7, CYP3A5, ADH1C, ADH1B	1.69E-04
hsa00350	Tyrosine metabolism	3	MAOB, ADH1C, ADH1B	1.54E-03

Notes: KEGG: Kyoto Encyclopedia of Genes and Genomes

### Validation of the miR-221-3p target genes in the drug metabolism-cytochrome P450 signaling pathway

**Fig. 12:**
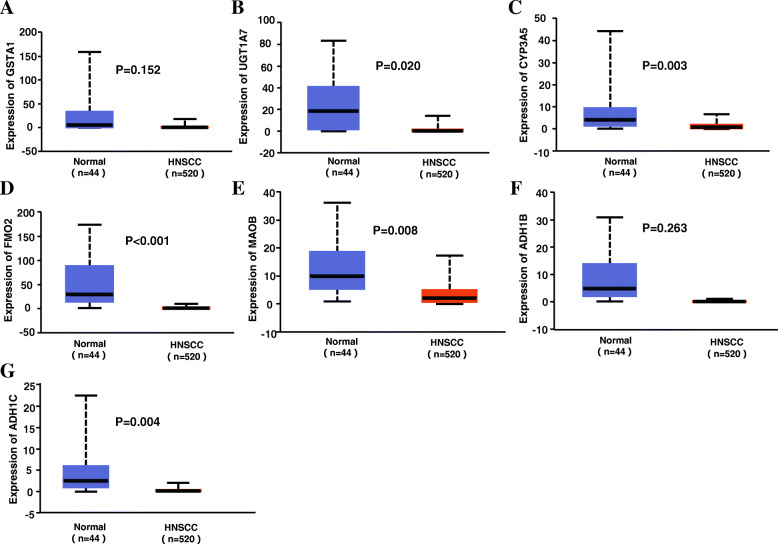
The expression levels of miR-221-3p target genes in HNSCC. **a**-**g** represents the expression of the following genes: GSTA1, UGT1A7, CYP3A5, FMO2, MAOB, ADH1C, ADH1B

**Fig. 13:**
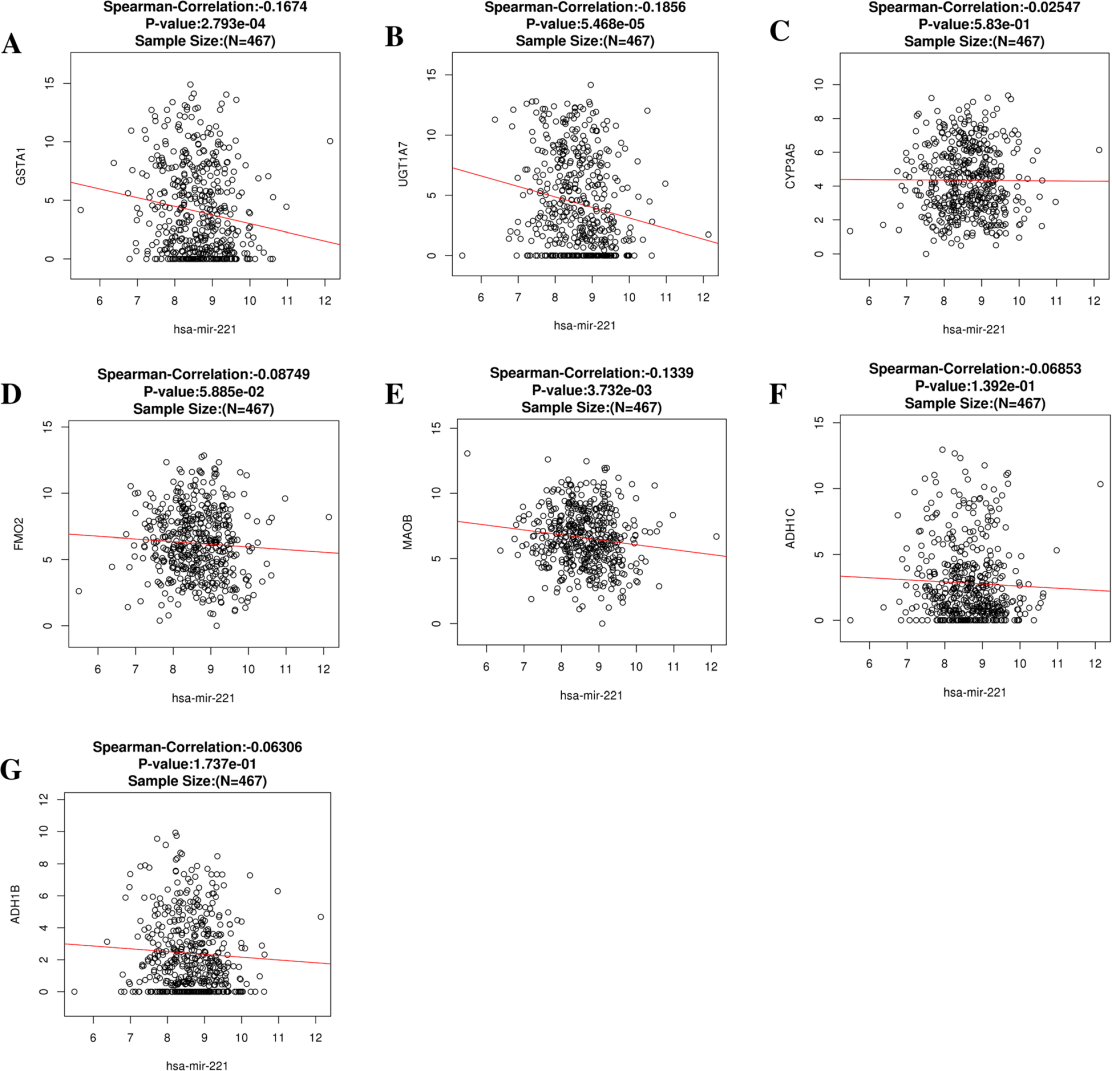
Spearman analysis. **a**-**g** show the association between miR-221-3p expression levels and the seven identified target genes involved in the Drug metabolism - cytochrome P450 pathway

The expression values of each target gene are shown in Fig. 12, from which we learned that the 5 target genes (UGT1A7, CYP3A5, FMO2, MAOB and ADH1B) related to miR-221-3p were downregulated in the drug metabolism-cytochrome P450 pathway (*p* < 0.05). Spearman correlation analysis showed that GSTA1, UGT1A7 and MAOB, the target genes of the drug metabolism-cytochrome P450, were correlated with miR-221-3p in HNSCC (p < 0.05, Fig.13).

## Discussion

The drug metabolism-cytochrome P450 signaling pathway is an essential signal transduction pathway in cells. It is an important biological function of cell survival, proliferation and apoptosis. For example, Liping Wang and his colleagues suggest [33] that the inhibition of CYP3A5 in cytochrome P450 drug metabolism can drive the migration, proliferation, and invasion of HNSCC cells. Dongfang Wang et al. showed that cytochrome P450 is correlated with the overall survival and vascular invasion of hepatocellular carcinoma (HCC) patients. Additionally, inhibiting the P450 pathway in drug metabolism-cytochrome can increase the efficacy of HNSCC [34]. Among the target genes of miR-221-3p, MAOB and UGT1A7 are associated with the miR-221-3p and drug-cytochrome P450 signaling pathway and are downregulated in HNSCC (p < 0.05). MAOB is regarded as a novel biomarker for accurate prostate cancer diagnosis and treatment [35]. In addition, a large number of valuable epidemiological studies have shown that UGT1A7 affects individuals’ susceptibility to various cancers, such as pancreatic cancer [36] and gastrointestinal carcinomas [37]. Our study suggests that miR-221-3p may play an important role as a gene that promotes the development of HNSCC by reducing the expression of the MAOB and UGT1A7 pathways in HNSCC.

Understanding the pathogenesis of HNSCC and identifying new gene therapy programs have been the focus of recent studies. Many recent studies have reported that miRNAs with different expression patterns in different tumors [38–42] control the progression of tumors. For example, miR-221-3p has been regarded as a tumor biomarker that can be used to assess the clinical prognosis of breast cancer [15]. In healthy humans, it has been shown that miR-221-3p plays a role in the process of vascular proliferation, while the tumor promoter miR-221-3p regulates the apoptosis of tumor cells. The current study focused on the expression pattern of miR-221-3p in HNSCC, identifying the exact target genes of this miRNA and its biological mechanism of action through bioinformatics analysis. The biological pathways relevant to the actions of miR-221-3p will guide further understanding of the mechanisms of HNSCC.

Of course, the limitations of our study should also be mentioned. On the one hand, this study is restricted in that the analysis of differential miRNAs was only based on HNSCC and noncancerous tissues, and other samples (e.g., blood) were not assessed. On the other hand, the target genes of miR-221-3p have not been experimentally confirmed, and our conclusions will need to be confirmed by clinical or molecular biological methods in the future. In addition, only English studies were used for the basis of our meta-analysis, which did not include other potentially relevant studies that were in other languages.

## Conclusion

In our study, we found that miR-221-3p levels were apparently upregulated in HNSCC compared to normal tissues. MAOB and UGT1A7 are potentially important targets of miR-221-3p. MiR-221-3p may be used as a noninvasive and hypersensitive biomarker for the diagnosis of HNSCC and is an extremely important gene locus involved in the process of the deterioration and eventual tumorigenesis of HNSCC. Finally, the biological pathways relevant to the actions of miR-221-3p will provide insights into its potential molecular mechanisms in HNSCC. Larger-scale studies will be needed to validate its diagnostic promise. Hopefully, additional work will validate its usefulness as a target for future clinical research.

## Data Availability

All data generated or analyzed during this study are included in this published article.

## Abbreviations

HNSCC: Head and neck squamous cell carcinoma
miR: microRNA
PPI: protein-protein interaction
GO: Gene Ontology
KEGG: Kyoto Encyclopedia of Genes and Genomes
GEO: Gene Expression Omnibus
TCGA: The cancer genome atlas
DAVID: Database for Annotation, Visualization and Integrated Discovery
BiNGO: Biological Networks Gene Oncology tool
BP: biological process
CC: cellular component
MF: molecular function
